# Supplementary material for: A New Induction Method for the Controlled Differentiation of Human-Induced Pluripotent Stem Cells Using Frozen Sections
Source: Cells. 2021 Oct 21;10(11):2827. doi: 10.3390/cells10112827 (PMC8616214; doi:10.3390/cells10112827)
Supplement: Supplementary file 1 [file cells-10-02827-s001.zip › cells-1417824-supplement - Copy.pdf]

Supplementary Information

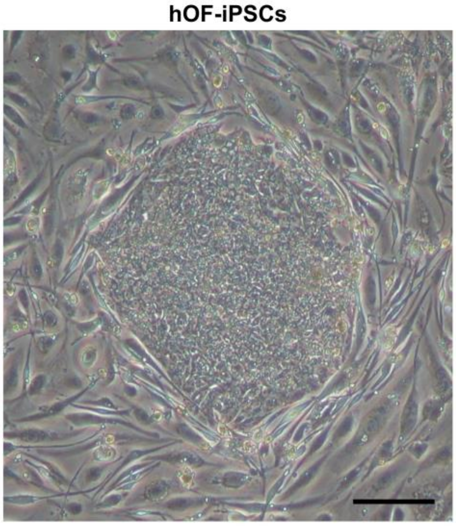

Figure S1. Phase-contrast microscopy of hOF-iPS on SNL feeder cells generated from the oral mucosa. These cells were generated from the isolated oral mucosa fibroblasts through the retroviral gene transfer of OCT3/4, SOX2, c-MYC and KLF4 [14]. Reprogramed cells showed ES-like morphology. Scale bars: 100 μm.

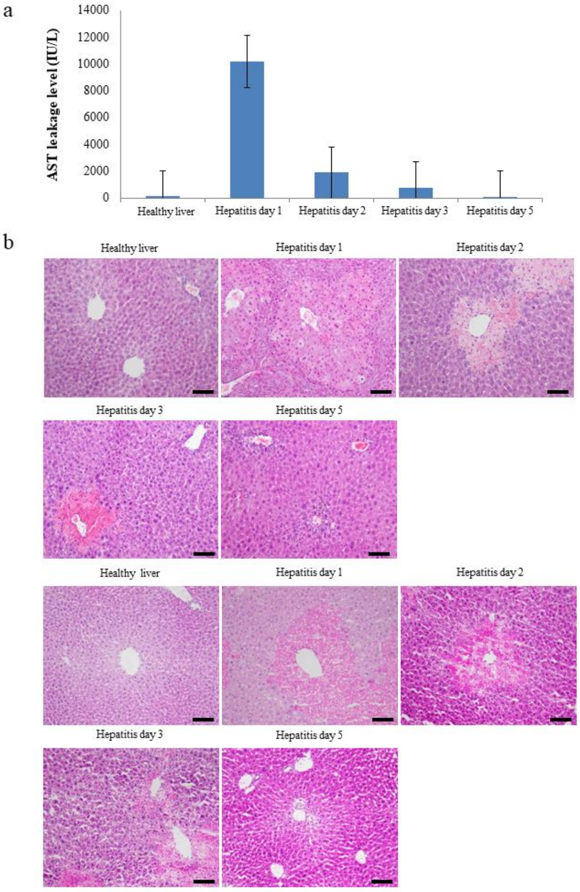

Figure S2. Histology of the frozen sections used to induce the differentiation of iPSCs. Acute hepatitis was induced by an intraperitoneal injection of 1 mL/kg carbon tetrachloride in 6-week-old male ICR mice. (a) Aspartate aminotransferase (AST) leakage levels in serum. One day after the induction of hepatitis, the AST leakage level increased. (b) Histology of the livers from healthy and hepatitis-induced mice. In healthy livers, normal hepatocytes were observed around the central vein. One day after the induction of hepatitis, we observed a toxic turbidity of the cytoplasm and swelling of hepatocytes around the central vein. Further, two days after the induction of hepatitis, we observed necrosis of edematous hepatocytes; three days after the induction of hepatitis, we observed regenerative alterations in bleeding around the central vein and cell mitosis surrounding necrotic hepatocytes; five days after the induction of hepatitis, we observed restoration of the healthy tissue appearance with the disappearance of necrotic hepatocytes. Thus, hepatocytes showed recovery from acute drug-induced hepatitis after five days. In this study, we used the frozen sections of healthy and injured livers on days 1, 2, 3, and 5. Scale bars: 100 μm.

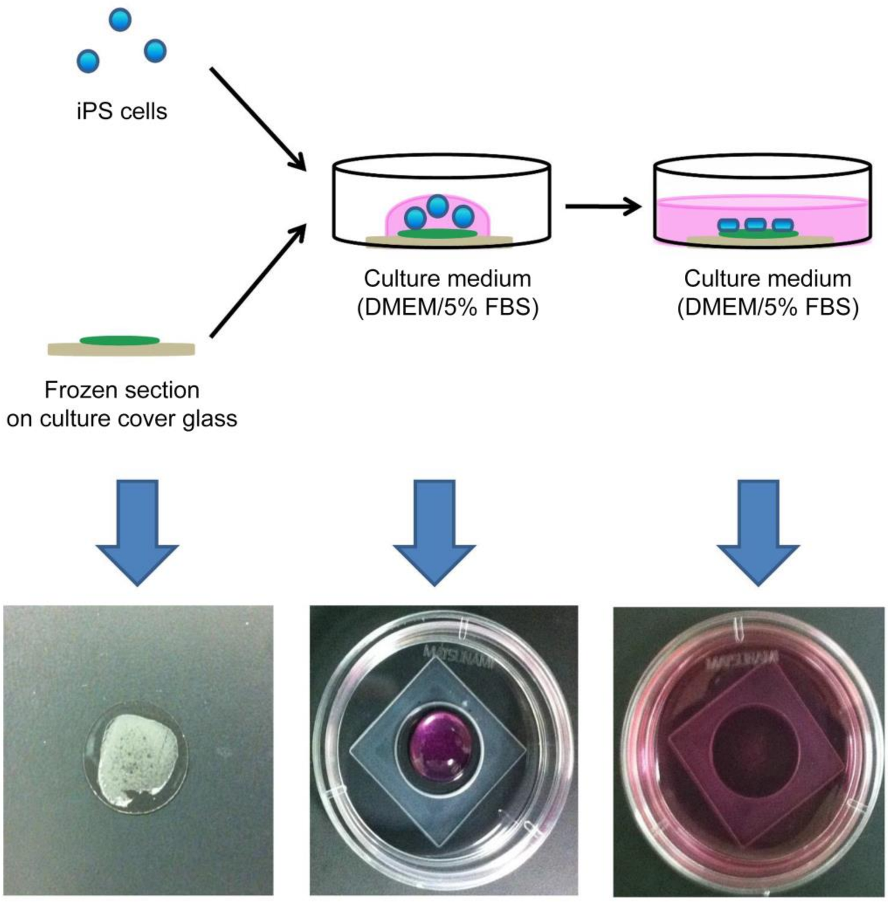

Figure S3. Method of culturing iPSCs on the frozen sections to induce differentiation. Frozen tissues were cut at 6-µm thickness and placed on round cover glasses (13 mm diameter) for cell culture. After iPSCs were cultured following the reported protocol, cells were collected and suspended in DMEM containing 5% fetal bovine serum (FBS). Next, iPSCs were seeded on the frozen sections as shown in the diagram. iPSCs were allowed to adhere to the frozen section for one day. Next, we added 2 mL DMEM containing 5% FBS. The medium was changed every three days, and cells were cultured for nine days.

	control 1	Healthy liver	Hepatitis day 1	Hepatitis day 2	Hepatitis day 3	Hepatitis day 5
NANOG	+	-	-	-	-	-
OCT3/4	+	-	-	-	-	-
SOX17	+	+	-	+	-	-
FOXA2	+	+	-	-	+	-
AFP	+	+	+	-	-	+
AAT	+	+	+	-	-	+
ALB	+	+	-	-	-	+
CYP3A4	-	-	-	-	-	-
NES	+	-	-	-	-	-
MBP	-	-	-	-	-	-
CNP	+	-	-	-	-	-
GFAP	+	-	-	-	-	-
NF200	-	-	-	-	-	-
TUJ1	+	-	-	-	-	-

Figure S4. Expression of various markers in the cultured hOF-iPSCs. RT–PCR was performed to assess the gene expression of hepatocyte-differentiated (*SOX17, FOXA2, AFP, AAT, ALB*, and *CYP3A4*), neuron-differentiated (*NES, MBP, CNP, GFAP, NF200,* and *TUJ1*), and undifferentiated (*NANOG* and *OCT3/4*) cell markers. Control hOF-iPSCs highly expressed undifferentiated cell markers, and a few hepatocyte- and neuron-differentiated markers were expressed at random. hOF-iPSCs cultured on the normal livers and livers with hepatitis highly expressed hepatocyte-differentiated markers, and undifferentiated cell markers and neuron-differentiated markers were not expressed.

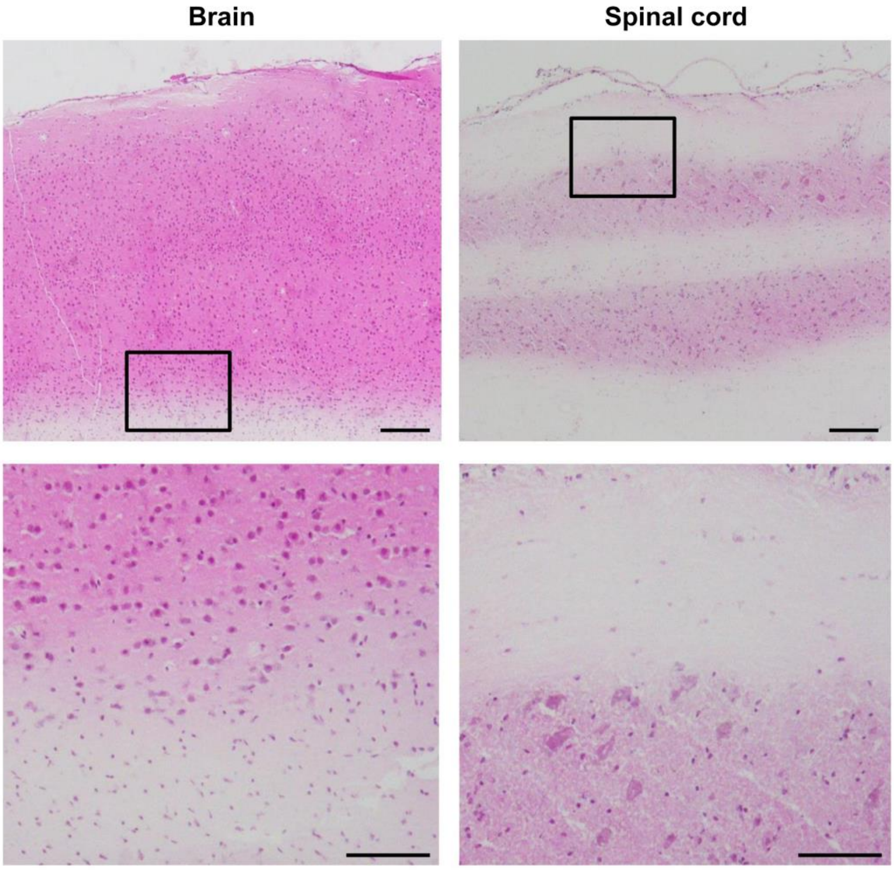

Figure S5. Histology of the frozen sections used to culture iPSCs. Histology of the sections of the brain and spinal cord taken from 6-week-old ICR mice. The cerebral cortex and medulla, including various neural cells, were observed in the sections of the brain. White and gray matter were observed in the sections of the spinal cord. Scale bars: 100 μm.

	control 1	Brain	Spinal cord
NANOG	+	-	-
OCT3/4	+	-	-
SOX17	+	-	-
FOXA2	+	-	-
AFP	+	-	-
AAT	+	-	-
ALB	+	-	-
CYP3A4	-	-	-
NES	+	-	-
MBP	-	+	-
CNP	+	+	+
GFAP	+	+	+
NF200	-	-	-
TUJ1	+	-	-

Figure S6. Expression of various markers on the cultured hOF-iPSCs**.** RT–PCR was used to assess the gene expression of hepatocyte-differentiated (*SOX17, FOXA2, AFP, AAT, ALB*, and *CYP3A4*), neuron-differentiated (*NES, MBP, CNP, GFAP, NF200,* and *TUJ1*), and undifferentiated (*NANOG* and *OCT3/4*) cell markers. Control hOF-iPSCs highly expressed undifferentiated cell markers, and a few hepatocyte- and neuron-differentiated markers were expressed at random. hOF-iPSCs cultured on the sections of the brain and spinal cord highly expressed neuron-differentiated markers. Undifferentiated cell markers and hepatocyte-differentiated markers were not expressed.

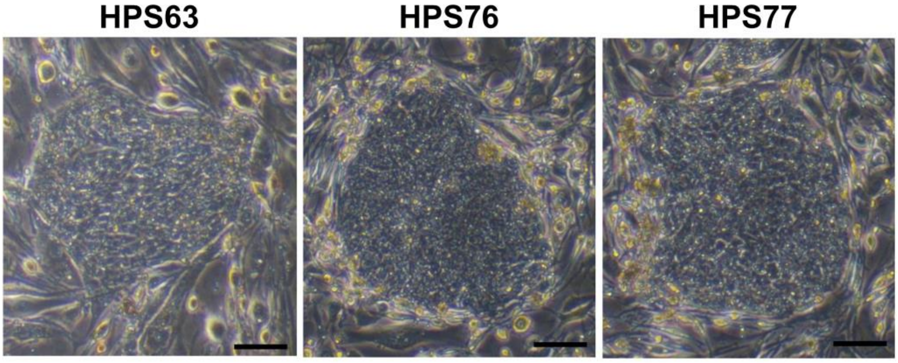

Figure S7. Phase-contrast microscopy of the iPSC colonies (HPS63, HPS76, and HPS77) obtained from the RIKEN Cell Bank and grown on SNL feeder cells generated from various somatic cells. HPS63 was generated from the isolated human skin fibroblasts through the retroviral gene transfer of OCT3/4, SOX2, c-MYC, and KLF4, which are the same factors as hOF-iPSCs [36]. HPS76 was generated from the isolated human skin fibroblasts through the episomal gene transfer of OCT3/4, SOX2, L-MYC, KLF4, Lin28, and shRNA against p53 [37]. HPS77 was generated from isolated human dental pulp cells through the episomal gene transfer of OCT3/4, SOX2, L-MYC, KLF4, Lin28, and shRNA against p53 [38]. Reprogramed cells showed ES-like morphology. Scale bars: 100 μm.

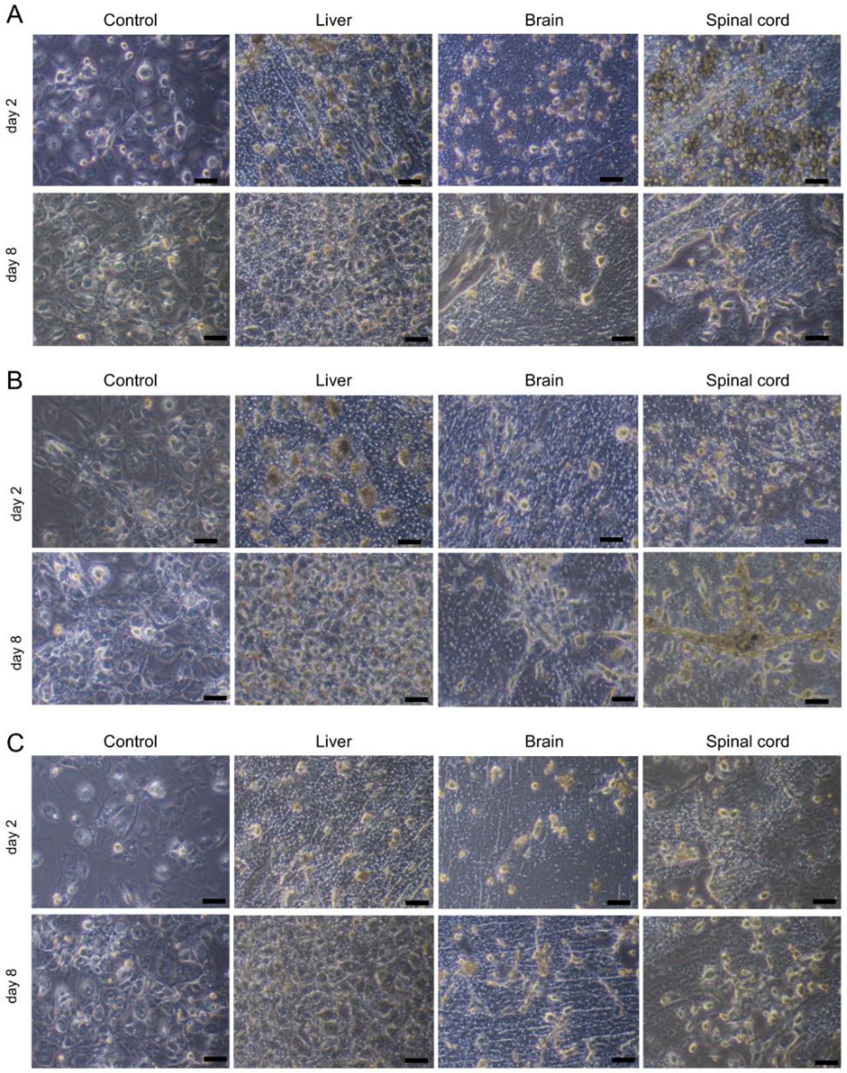

Figure S8. Phase-contrast microscopy of iPSCs (HPS63, HPS76 and HPS77) cultured on cover glasses (controls) and frozen sections of the liver, brain and spinal cord. (A) HPS63. On day 2, HPS63 cultured on cover glasses and frozen sections was loosely spread. On day 8, HPS63 cultured on cover glasses showed various morphological changes, whereas HPS63 cultured on frozen sections of the liver showed comparatively large and polygonal morphological changes resembling hepatocytes. HPS63 cultured on frozen sections of the brain and spinal cord developed neuronal morphological traits. The cytoplasm of flat HPS63 was retracted toward the nucleus, forming a contracted multipolar cell body with membranous, process-like peripheral extensions. Cell bodies became increasingly spherical and refractile, exhibiting a typical neuronal perikaryal appearance. These findings are similar to those obtained with hOF-iPS. (B) HPS76 and (C) HPS77 showed similar patterns. Scale bars: 100 µm.

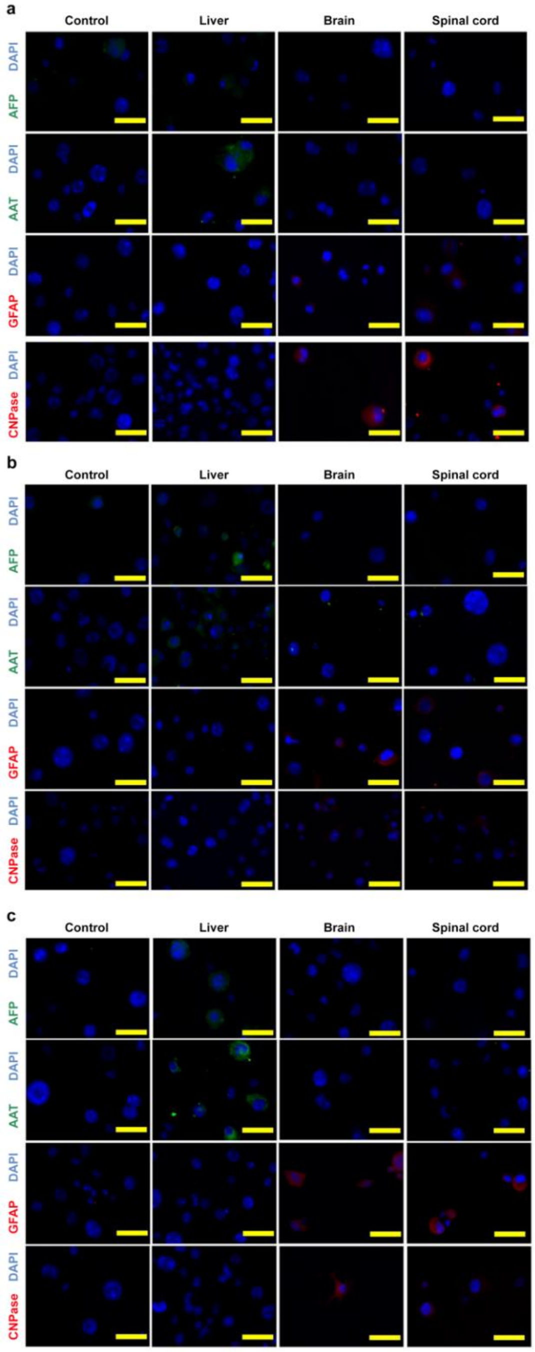

Figure S9. Immunocytochemical analysis of hepatocyte-differentiated (AFP and AAT) and neuron-differentiated (GFAP and CNPase) markers in iPSCs. DAPI was used as a counterstain. (a) HPS63 cultured on the liver sections showed high AFP and AAT expression, whereas neuron-differentiated markers, i.e. GFAP and CNPase, were highly expressed in HPS63 cultured on sections of the brain and spinal cord. These findings are similar to those obtained with hOF-iPS. (b) HPS76 and (c) HPS77 showed similar patterns. Scale bars: 100 μm.
